# Supplementary figures and images for: Mechanisms of sterilizing immunity provided by an HIV-1 neutralizing antibody against mucosal infection
Source: PLoS Pathog. 2024 Dec 26;20(12):e1012777. doi: 10.1371/journal.ppat.1012777 (PMC11670951; doi:10.1371/journal.ppat.1012777)

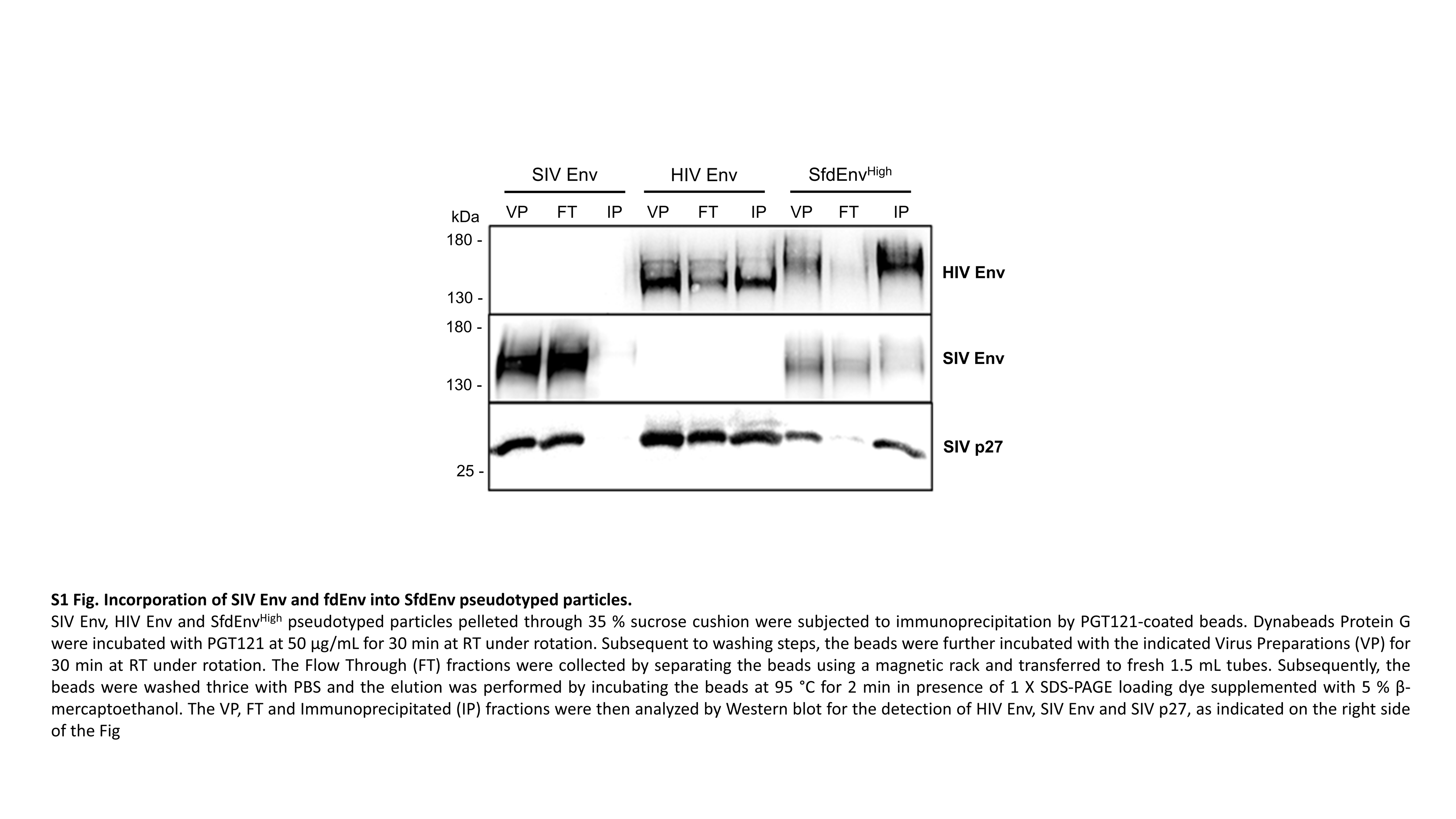

Supplement: S1 Fig — SIV Env, HIV Env and SfdEnvHigh pseudotyped particles pelleted through 35% sucrose cushion were subjected to immunoprecipitation by PGT121-coated beads. Dynabeads Protein G were incubated with PGT121 at 50 μg/mL for 30 min at RT under rotation. Subsequent to washing steps, the beads were further incubated with the indicated Virus Preparations (VP) for 30 min at RT under rotation. The Flow Through (FT) fractions were collected by separating the beads using a magnetic rack and transferred to fresh 1.5 mL tubes. Subsequently, the beads were washed thrice with PBS and the elution was performed by incubating the beads at 95°C for 2 min in presence of 1 X SDS-PAGE loading dye supplemented with 5% β-mercaptoethanol. The VP, FT and Immunoprecipitated (IP) fractions were then analyzed by Western blot for the detection of HIV Env, SIV Env and SIV p27, as indicated on the right side of the Fig. (TIF) [file ppat.1012777.s001.tif]

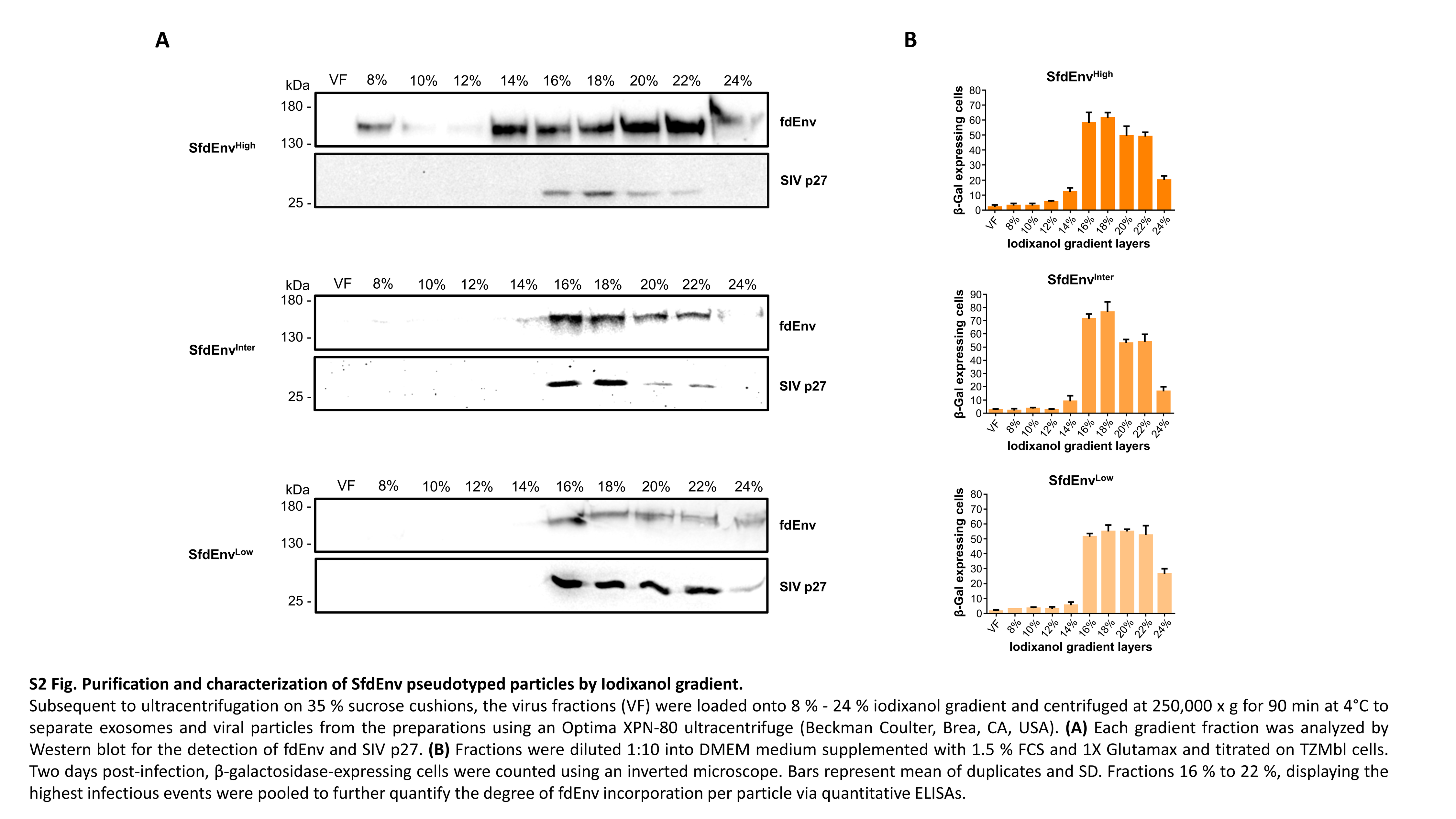

Supplement: S2 Fig — Subsequent to ultracentrifugation on 35% sucrose cushions, the virus fractions (VF) were loaded onto 8% - 24% iodixanol gradient and centrifuged at 250,000 x g for 90 min at 4°C to separate exosomes and viral particles from the preparations using an Optima XPN-80 ultracentrifuge (Beckman Coulter, Brea, CA, USA). (A) Each gradient fraction was analyzed by Western blot for the detection of fdEnv and SIV p27. (B) Fractions were diluted 1:10 into DMEM medium supplemented with 1.5% FCS and 1X Glutamax and titrated on TZMbl cells. Two days post-infection, β-galactosidase-expressing cells were counted using an inverted microscope. Bars represent mean of duplicates and SD. Fractions 16% to 22%, displaying the highest infectious events were pooled to further quantify the degree of fdEnv incorporation per particle via quantitative ELISAs. (TIF) [file ppat.1012777.s002.tif]

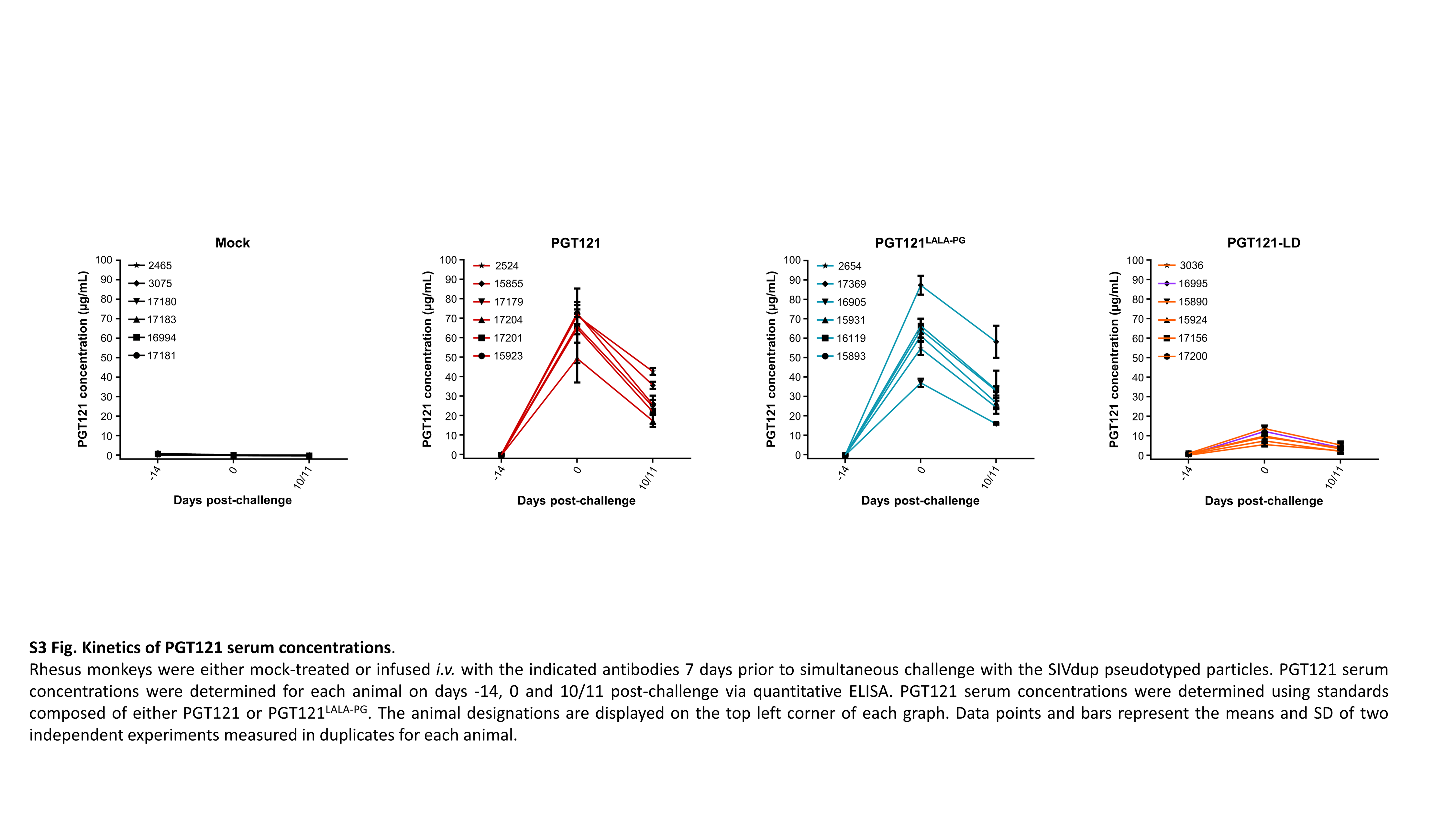

Supplement: S3 Fig — Rhesus monkeys were either mock-treated or infused i.v. with the indicated antibodies 7 days prior to simultaneous challenge with the SIVdup pseudotyped particles. PGT121 serum concentrations were determined for each animal on days -14, 0 and 10/11 post-challenge via quantitative ELISA. PGT121 serum concentrations were determined using standards composed of either PGT121 or PGT121LALA-PG. The animal designations are displayed on the top left corner of each graph. Data points and bars represent the means and SD of two independent experiments measured in duplicates for each animal. (TIF) [file ppat.1012777.s003.tif]

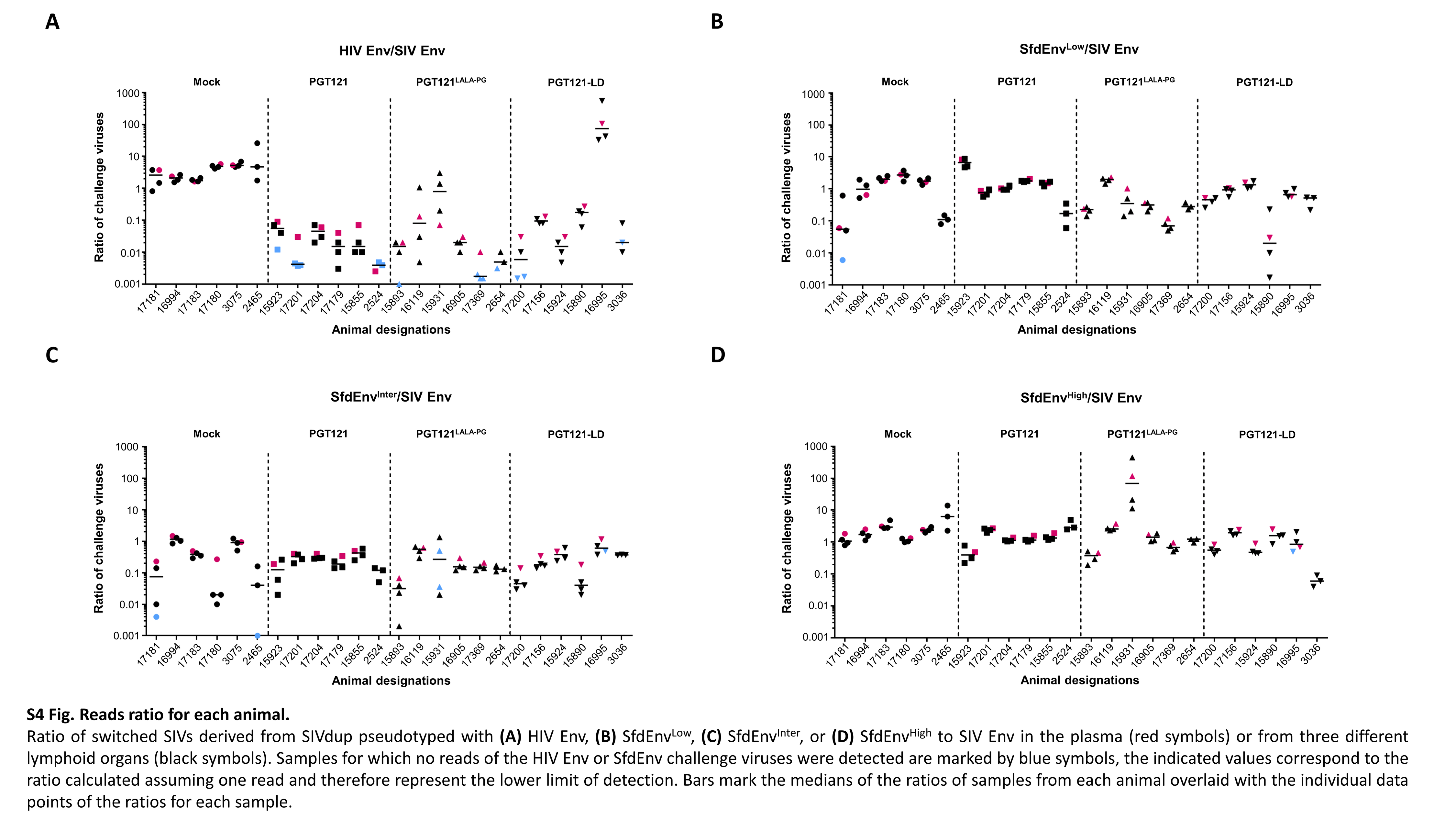

Supplement: S4 Fig — Ratio of switched SIVs derived from SIVdup pseudotyped with (A) HIV Env, (B) SfdEnvLow, (C) SfdEnvInter, or (D) SfdEnvHigh to SIV Env in the plasma (red symbols) or from three different lymphoid organs (black symbols). Samples for which no reads of the HIV Env or SfdEnv challenge viruses were detected are marked by blue symbols, the indicated values correspond to the ratio calculated assuming one read and therefore represent the lower limit of detection. Bars mark the medians of the ratios of samples from each animal overlaid with the individual data points of the ratios for each sample. (TIF) [file ppat.1012777.s004.tif]
