# Supplementary material for: Mechanisms of sterilizing immunity provided by an HIV-1 neutralizing antibody against mucosal infection
Source: PLoS Pathog. 2024 Dec 26;20(12):e1012777. doi: 10.1371/journal.ppat.1012777 (PMC11670951; doi:10.1371/journal.ppat.1012777)
Supplement: S1 Table — (DOCX) [file ppat.1012777.s005.docx]

|  |  |  | **Lymph nodes** | | |
| --- | --- | --- | --- | --- | --- |
| **Animals** | **Tags (Env)** | **Plasma** | **Mesenteric** | **Inguinal** | **Submandibular** |
| **Mock** |  |  |  |  |  |
| 17181 | CCT-CGA (HIV Env) | 6231 | 528 | 174 | 246 |
|  | CCT-AGG (SIV Env) | 1676 | 141 | 216 | 167 |
|  | TAG (SfdEnv^High^) | 3067 | 111 | 254 | 158 |
|  | CCT (SfdEnv^Inter^) | 390 | 1 | 30 | 0 |
|  | AAC-AGA (SfdEnv^Low^) | 97 | 7 | 134 | 0 |
| 16994 | CCT-CGA (HIV Env) | 7630 | 217 | 421 | 241 |
|  | CCT-AGG (SIV Env) | 3217 | 118 | 160 | 155 |
|  | TAG (SfdEnv^High^) | 8038 | 184 | 301 | 174 |
|  | CCT (SfdEnv^Inter^) | 4772 | 122 | 208 | 132 |
|  | AAC-AGA (SfdEnv^Low^) | 2093 | 61 | 312 | 201 |
| 17183 | CCT-CGA (HIV Env) | 5209 | 280 | 277 | 298 |
|  | CCT-AGG (SIV Env) | 3214 | 154 | 132 | 182 |
|  | TAG (SfdEnv^High^) | 9938 | 429 | 361 | 873 |
|  | CCT (SfdEnv^Inter^) | 1560 | 64 | 38 | 63 |
|  | AAC-AGA (SfdEnv^Low^) | 5775 | 272 | 335 | 401 |
| 17180 | CCT-CGA (HIV Env) | 9114 | 502 | 539 | 564 |
|  | CCT-AGG (SIV Env) | 1594 | 108 | 106 | 138 |
|  | TAG (SfdEnv^High^) | 2108 | 139 | 104 | 144 |
|  | CCT (SfdEnv^Inter^) | 435 | 1 | 2 | 3 |
|  | AAC-AGA (SfdEnv^Low^) | 4493 | 405 | 280 | 238 |
| 3075 | CCT-CGA (HIV Env) | 11347 | 591 | 652 | 591 |
|  | CCT-AGG (SIV Env) | 2432 | 116 | 95 | 116 |
|  | TAG (SfdEnv^High^) | 5920 | 269 | 282 | 269 |
|  | CCT (SfdEnv^Inter^) | 2297 | 103 | 116 | 103 |
|  | AAC-AGA (SfdEnv^Low^) | 3980 | 217 | 205 | 217 |
| 2465 | CCT-CGA (HIV Env) | nd | 244 | 128 | 591 |
|  | CCT-AGG (SIV Env) | nd | 52 | 73 | 116 |
|  | TAG (SfdEnv^High^) | nd | 326 | 165 | 269 |
|  | CCT (SfdEnv^Inter^) | nd | 2 | 12 | 103 |
|  | AAC-AGA (SfdEnv^Low^) | nd | 8 | 6 | 217 |

**S1 Table. Number of reads derived from the different challenge viruses for the mock-treated animals.**

nd = not determined
