# Supplementary material for: Mechanisms of sterilizing immunity provided by an HIV-1 neutralizing antibody against mucosal infection
Source: PLoS Pathog. 2024 Dec 26;20(12):e1012777. doi: 10.1371/journal.ppat.1012777 (PMC11670951; doi:10.1371/journal.ppat.1012777)
Supplement: S2 Table — (DOCX) [file ppat.1012777.s006.docx]

|  |  |  | **Lymph nodes** | | |
| --- | --- | --- | --- | --- | --- |
| **Animals** | **Tags (Env)** | **Plasma** | **Mesenteric** | **Inguinal** | **Submandibular** |
| **PGT121** |  |  |  |  |  |
| 15923 | CCT-CGA (HIV Env) | 157 | 0 | 9 | 5 |
|  | CCT-AGG (SIV Env) | 1689 | 82 | 128 | 121 |
|  | TAG (SfdEnv^High^) | 819 | 18 | 100 | 38 |
|  | CCT (SfdEnv^Inter^) | 317 | 5 | 33 | 2 |
|  | AAC-AGA (SfdEnv^Low^) | 13900 | 712 | 605 | 615 |
| 17201 | CCT-CGA (HIV Env) | 180 | 0 | 0 | 0 |
|  | CCT-AGG (SIV Env) | 5367 | 272 | 225 | 264 |
|  | TAG (SfdEnv^High^) | 14353 | 719 | 533 | 514 |
|  | CCT (SfdEnv^Inter^) | 2161 | 104 | 44 | 72 |
|  | AAC-AGA (SfdEnv^Low^) | 4651 | 260 | 128 | 177 |
| 17204 | CCT-CGA (HIV Env) | 534 | 8 | 8 | 7 |
|  | CCT-AGG (SIV Env) | 8247 | 262 | 123 | 439 |
|  | TAG (SfdEnv^High^) | 11347 | 295 | 137 | 457 |
|  | CCT (SfdEnv^Inter^) | 3267 | 77 | 35 | 131 |
|  | AAC-AGA (SfdEnv^Low^) | 8479 | 331 | 117 | 414 |
| 17179 | CCT-CGA (HIV Env) | 218 | 4 | 1 | 3 |
|  | CCT-AGG (SIV Env) | 5058 | 246 | 304 | 332 |
|  | TAG (SfdEnv^High^) | 7971 | 288 | 342 | 343 |
|  | CCT (SfdEnv^Inter^) | 1726 | 57 | 47 | 45 |
|  | AAC-AGA (SfdEnv^Low^) | 10382 | 444 | 502 | 585 |
| 15855 | CCT-CGA (HIV Env) | 522 | 3 | 4 | 2 |
|  | CCT-AGG (SIV Env) | 7943 | 190 | 332 | 237 |
|  | TAG (SfdEnv^High^) | 14957 | 267 | 389 | 298 |
|  | CCT (SfdEnv^Inter^) | 3910 | 110 | 84 | 85 |
|  | AAC-AGA (SfdEnv^Low^) | 11949 | 320 | 398 | 373 |
| 2524 | CCT-CGA (HIV Env) | nd | 1 | 0 | 0 |
|  | CCT-AGG (SIV Env) | nd | 405 | 256 | 210 |
|  | TAG (SfdEnv^High^) | nd | 1003 | 729 | 1040 |
|  | CCT (SfdEnv^Inter^) | nd | 57 | 14 | 26 |
|  | AAC-AGA (SfdEnv^Low^) | nd | 26 | 43 | 74 |

**S2 Table. Number of reads derived from the different challenge viruses for the PGT121-treated animals.**

nd = not determined
