# Supplementary material for: Mechanisms of sterilizing immunity provided by an HIV-1 neutralizing antibody against mucosal infection
Source: PLoS Pathog. 2024 Dec 26;20(12):e1012777. doi: 10.1371/journal.ppat.1012777 (PMC11670951; doi:10.1371/journal.ppat.1012777)
Supplement: S3 Table — (DOCX) [file ppat.1012777.s007.docx]

|  |  |  | **Lymph nodes** | | |
| --- | --- | --- | --- | --- | --- |
| **Animals** | **Tags (Env)** | **Plasma** | **Mesenteric** | **Inguinal** | **Submandibular** |
| **PGT121^LALA-PG^** |  |  |  |  |  |
| 15893 | CCT-CGA (HIV Env) | 137 | 6 | 9 | 0 |
|  | CCT-AGG (SIV Env) | 8704 | 734 | 451 | 1368 |
|  | TAG (SfdEnv^High^) | 3974 | 216 | 229 | 262 |
|  | CCT (SfdEnv^Inter^) | 579 | 17 | 18 | 3 |
|  | AAC-AGA (SfdEnv^Low^) | 2107 | 195 | 93 | 192 |
| 16119 | CCT-CGA (HIV Env) | 273 | 6 | 1 | 113 |
|  | CCT-AGG (SIV Env) | 2173 | 172 | 207 | 107 |
|  | TAG (SfdEnv^High^) | 8127 | 430 | 548 | 246 |
|  | CCT (SfdEnv^Inter^) | 1357 | 115 | 59 | 52 |
|  | AAC-AGA (SfdEnv^Low^) | 4959 | 249 | 434 | 201 |
| 15931 | CCT-CGA (HIV Env) | 260 | 6 | 2 | 9 |
|  | CCT-AGG (SIV Env) | 189 | 2 | 28 | 45 |
|  | TAG (SfdEnv^High^) | 21958 | 893 | 591 | 510 |
|  | CCT (SfdEnv^Inter^) | 253 | 0 | 0 | 1 |
|  | AAC-AGA (SfdEnv^Low^) | 194 | 1 | 4 | 9 |
| 16905 | CCT-CGA (HIV Env) | 271 | 8 | 7 | 7 |
|  | CCT-AGG (SIV Env) | 7897 | 369 | 520 | 284 |
|  | TAG (SfdEnv^High^) | 13514 | 388 | 584 | 529 |
|  | CCT (SfdEnv^Inter^) | 2268 | 58 | 77 | 35 |
|  | AAC-AGA (SfdEnv^Low^) | 2821 | 137 | 140 | 57 |
| 17369 | CCT-CGA (HIV Env) | 147 | 0 | 0 | 0 |
|  | CCT-AGG (SIV Env) | 10106 | 509 | 686 | 648 |
|  | TAG (SfdEnv^High^) | 9718 | 305 | 347 | 488 |
|  | CCT (SfdEnv^Inter^) | 2072 | 60 | 107 | 88 |
|  | AAC-AGA (SfdEnv^Low^) | 1181 | 43 | 37 | 39 |
| 2654 | CCT-CGA (HIV Env) | nd | 2 | 3 | 0 |
|  | CCT-AGG (SIV Env) | nd | 407 | 516 | 326 |
|  | TAG (SfdEnv^High^) | nd | 390 | 633 | 401 |
|  | CCT (SfdEnv^Inter^) | nd | 70 | 65 | 35 |
|  | AAC-AGA (SfdEnv^Low^) | nd | 113 | 193 | 74 |

**S3 Table. Number of reads derived from the different challenge viruses for the PGT121^LALA-PG^-treated animals.**

nd = not determined
