# Supplementary material for: Mechanisms of sterilizing immunity provided by an HIV-1 neutralizing antibody against mucosal infection
Source: PLoS Pathog. 2024 Dec 26;20(12):e1012777. doi: 10.1371/journal.ppat.1012777 (PMC11670951; doi:10.1371/journal.ppat.1012777)
Supplement: S4 Table — (DOCX) [file ppat.1012777.s008.docx]

|  |  |  | **Lymph nodes** | | |
| --- | --- | --- | --- | --- | --- |
| **Animals** | **Tags (Env)** | **Plasma** | **Mesenteric** | **Inguinal** | **Submandibular** |
| **PGT121-LD** |  |  |  |  |  |
| 17200 | CCT-CGA (HIV Env) | 157 | 0 | 4 | 0 |
|  | CCT-AGG (SIV Env) | 5310 | 661 | 584 | 598 |
|  | TAG (SfdEnv^High^) | 4444 | 345 | 347 | 247 |
|  | CCT (SfdEnv^Inter^) | 721 | 31 | 24 | 16 |
|  | AAC-AGA (SfdEnv^Low^) | 2771 | 341 | 234 | 156 |
| 17156 | CCT-CGA (HIV Env) | 837 | 25 | 32 | 34 |
|  | CCT-AGG (SIV Env) | 6258 | 234 | 395 | 453 |
|  | TAG (SfdEnv^High^) | 15123 | 376 | 685 | 1000 |
|  | CCT (SfdEnv^Inter^) | 2141 | 33 | 76 | 76 |
|  | AAC-AGA (SfdEnv^Low^) | 6523 | 131 | 351 | 445 |
| 15924 | CCT-CGA (HIV Env) | 174 | 1 | 3 | 4 |
|  | CCT-AGG (SIV Env) | 5147 | 168 | 642 | 263 |
|  | TAG (SfdEnv^High^) | 4615 | 84 | 289 | 118 |
|  | CCT (SfdEnv^Inter^) | 2410 | 104 | 157 | 75 |
|  | AAC-AGA (SfdEnv^Low^) | 8145 | 297 | 666 | 297 |
| 15890 | CCT-CGA (HIV Env) | 2125 | 32 | 48 | 112 |
|  | CCT-AGG (SIV Env) | 7815 | 579 | 302 | 588 |
|  | TAG (SfdEnv^High^) | 19570 | 502 | 464 | 951 |
|  | CCT (SfdEnv^Inter^) | 1382 | 12 | 16 | 20 |
|  | AAC-AGA (SfdEnv^Low^) | 212 | 1 | 69 | 4 |
| 16995 | CCT-CGA (HIV Env) | 29350 | 1091 | 788 | 593 |
|  | CCT-AGG (SIV Env) | 277 | 2 | 24 | 14 |
|  | TAG (SfdEnv^High^) | 193 | 0 | 24 | 29 |
|  | CCT (SfdEnv^Inter^) | 322 | 0 | 9 | 10 |
|  | AAC-AGA (SfdEnv^Low^) | 161 | 2 | 18 | 8 |
| 3036 | CCT-CGA (HIV Env) | nd | 9 | 0 | 66 |
|  | CCT-AGG (SIV Env) | nd | 630 | 428 | 839 |
|  | TAG (SfdEnv^High^) | nd | 23 | 37 | 48 |
|  | CCT (SfdEnv^Inter^) | nd | 234 | 154 | 318 |
|  | AAC-AGA (SfdEnv^Low^) | nd | 136 | 223 | 456 |

**S4 Table. Number of reads derived from the different challenge viruses for the PGT121-LD-treated animals.**

nd = not determined
