# Supplementary material for: Mechanisms of sterilizing immunity provided by an HIV-1 neutralizing antibody against mucosal infection
Source: PLoS Pathog. 2024 Dec 26;20(12):e1012777. doi: 10.1371/journal.ppat.1012777 (PMC11670951; doi:10.1371/journal.ppat.1012777)
Supplement: S1 Text — (DOCX) [file ppat.1012777.s009.docx]

**Supporting Methods**

*Immunoprecipitation of SIVdup pseudotypes by PGT121*

To verify incorporation of both SIV Env and fdEnv into SIVdup particles, SIV Env, HIV Env and SfdEnv^High^ pseudotyped particles pelleted through 35 % sucrose cushions were resuspended in PBS and subjected to immunoprecipitation by PGT121 antibody. For that purpose, magnetic Dynabeads Protein G (Invitrogen, Waltham, MA, USA) were incubated for 30 min at RT under rotation with 200 μL of PGT121 antibody diluted at 50 μg/mL into PBS-0.05 % Tween20 (PBS-T). Following one wash with 500 μL PBS-T and one with 500 μL PBS, the PGT121-coated beads were resuspended with 50 μL of the respective SIVdup pseudotypes tested and incubated for 30 min at RT under constant rotation. The beads were placed on a magnetic rack and the supernatants were transferred to fresh 1.5 mL tubes to constitute the flow through fractions of the immunoprecipitation. Subsequently, the beads were washed thrice with 500 μL PBS, resuspended into 35 μL PBS and transferred to fresh 1.5 mL tubes containing 35 μL of 2X SDS-PAGE loading dye supplemented with 5 % β-mercaptoethanol. The same procedure was performed for the virus particle and flow-through fractions. All samples were boiled for 2 min at 95 °C on a heat-block and the beads from the immunoprecipitation fractions were separated on the magnetic rack. Co-immunoprecipitation of SIV Env and fdEnv were then analyzed by Western blot with anti-HIV-1 gp120 antibodies (BP1035, Acris, Herford, Germany), anti-SIVmac251 gp120 antibody (KK8, obtained through the NIH AIDS Research and Reagent Program from Dr. K. Kent) and anti-p24 antibody (183-H12-5C, obtained from the NIH AIDS Research and Reagent Program)

*Separation of exosomes and virus particles by iodixanol velocity gradients*

After purification of SfdEnv^Low^, SfdEnv^Inter^ and SfdEnv^High^ challenge viruses by ultracentrifugation on 35 % sucrose cushions, the particles were resuspended into 1 mL of PBS and loaded on top of iodixanol gradients consisting of 1 mL layers ranging from 8-24 % iodixanol with a 2% increment step. Following ultracentrifugation at 250,000 x g for 90 min at 4 °C using an Optima XPN-80 ultracentrifuge (Beckman Coulter, Brea, CA, USA), each layer was carefully collected by pipetting 1 mL from the top of the gradients, transferred to 1.5mL ultracentrifugation tubes and diluted with 500 µL PBS per layer. Subsequently, the pseudotyped particles were further ultracentrifuged for 2 h 30 min at 48,000 rpm at 4 °C using an Optima TLX ultracentrifuge (Beckman Coulter, Brea, CA, USA) and resuspended in 100 µL PBS. Each layer was analyzed by western blot for the detection of SIV p27 and fdEnv using anti-p24 antibody (183-H12-5C, obtained from the NIH AIDS Research and Reagent Program) and anti-HIV-1 gp120 antibodies (BP1035, Acris, Herford, Germany), respectively. Additionally, each layer was diluted 1:10 in DMEM medium supplemented with 1.5 % FCS; 1X Glutamax, and 50 µL were transferred in duplicates on TZMbl previously seeded the day before in flat-bottom 96-well plates. Following a 4 h incubation at 37 °C in a humidified incubator with 5 % CO_2_, 100 µL of complete DMEM medium was added into each well. After 48 h, the cells were washed with PBS, fixed with 0.5 % Glutaraldehyde for 10 min at RT, washed three additional times with PBS, and incubated for at least 2 h at 37 °C in the presence of X-Gal. Finally, the number of β-galactosidase-expressing cells per well was counted using an inverted microscope.

*Determination of PGT121 serum concentrations*

PGT121 and PGT121^LALA-PG^ serum concentrations were determined by quantitative ELISA in duplicates on day -14 prior to the challenge and on days 0 and 10/11 post-challenge for each animal. After saturation with 5 % powdered milk in PBS-T, HIV ConB gp120-coated high-binding 96-well plates (Greiner, Kremsünster, Austria) were incubated with the sera isolated from each monkey diluted at 1:12,500 into 2.5 % powdered milk in PBS-T. PGT121 binding was detected via polyclonal anti-human IgG antibody conjugated to HRP (Dianova, Hamburg, Germany) and chemiluminescence was measured by a Victor X4 multilable plate reader (Perkin Elmer, Hamburg, Germany) after the addition of ECL. To quantify PGT121 serum concentrations, the ELISAs were carried out against standards composed of either PGT121 or PGT121^LALA-PG^ antibodies.
