# Supplementary material for: Mechanisms of sterilizing immunity provided by an HIV-1 neutralizing antibody against mucosal infection
Source: PLoS Pathog. 2024 Dec 26;20(12):e1012777. doi: 10.1371/journal.ppat.1012777 (PMC11670951; doi:10.1371/journal.ppat.1012777)
Supplement: Attachment — Submitted filename: Response to Reviewers.pdf [file ppat.1012777.s011.pdf]

**Response to Reviewers` comments**

**Part I**
**Reviewer #1:**
Richel et al use an elegant SIV system to expand upon their work showing that Fc functions are not necessary for protective efficacy. The field has long discussed the levels of Env in relation to immune targeting and this work very nicely addresses that issue in respect to protection from different levels of challenge in macaques. The work is innovative and should be of interest to Plos Path readers. I have only minor comments.

1. The discussion seems unbalanced in that paragraphs describe how Fc functions may be useful (generally in rather contrived situations or using mice, generally with modest effects), yet this elegant study as well as multiple other well conducted primate studies with recent more potent Nab show that Fc functions are not needed. While it may be that non-nab exert some pressure on virus levels after infection has been established, this work along with other work of the authors and others, is convincing regarding the (lack of) role of non-nabs in prevention of infection.

**Authors:** The discussion has been extensively modified as suggested by the reviewer

2. Did the authors consider EM imaging of the SfdEnvLow, SfdEnvInter and SfdEnvHigh viruses for Env spikes. I think that would add to the characterisation.

**Authors:** We agree that this would be nice. However, accurately quantifying the number of trimers per virion by cryoelectronmicroscopy is methodologically challenging and beyond our capabilities. We would also like to point out, that determination of the number of spikes per virion for primary HIV isolates by Chertova *et. al.* (PMID: 11991960) is based on biochemical quantification of the ratio of Gag and Env proteins, which strongly correlate with cryoEM analysis (PMID: 16728975). Additionally, Zhu *et al.* demonstrated that the biochemical estimation of Env trimers per virion closely matched those measured using electron tomography (PMID: 14668432)

3. There are additional recent or relevant macaque papers that address this issue and are consistent with the authors data. PMID: 39198422 PMID: 34385004

**Authors:** Thanks for pointing out these interesting papers that are now cited in the revised manuscript.

---

**Part II – Major Issues: Key Experiments Required for Acceptance**

**Reviewer #2:**

1. The SIVdup system allows the authors to target the challenge virus at the beginning of the first replication cycle. Indeed, upon reverse transcription completion, the only Env being expressed is SIVEnv. Therefore, in their system, PGT121 can act only against viral particles during the first cycle, but not against infected cells. So, when the authors say that they explore "whether Fc-effector functions are relevant for providing sterilizing immunity against mucosal HIV infections" they must clearly specify which Fc-effector functions they refer to. They are certainly looking at virion opsonization, virolysis and perhaps ADCP, but certainly not ADCC which measures the lysis of the infected cells. In their system, the infected cells express SIVEnv and therefore it does not allow them to look at ADCC mediated by the HIV-1 Env-specific PGT121 Ab. This needs to be clarified throughout the manuscript otherwise it would be misleading. It is possible that ADCC plays a role at the early stages upon infection, when there is a limited number of infected cells but their system does not allow the authors to measure this.

**Authors:** We agree that our challenge virus system does not allow for the assessment of PGT121 ADCC activity, as the infected first cells should not display HIV Env on their surface. However, we would like to emphasize that a potential role of ADCC for sterilizing immunity also depends on the specific definition of sterilizing immunity. Therefore, we now define the use of sterilizing immunity in our manuscript as prevention of infection of the first cells by viruses present in the material the animals are exposed to and mention in the discussion section, that ADCC activity can not be assessed by our experimental set-up.

2. There is no such a thing as non-neutralizing binding site for PGT121. In the context of the current work, PGT121 binds to an HIV Env which is inactive. I think that this is what the authors try to refer to when they say that it is non-neutralizing. However, whenever PGT121 interacts with its "binding site" it will neutralize if the HIV Env is fusion competent. This needs to be corrected otherwise it will confuse the reader.

**Authors:** We realize that our phrasing can be misunderstood. Therefore, we now rephrase relevant sections of the manuscript by using the term non-neutralizing binding sites “on the virion” or “viral particle”.

---

## Part III – Minor Issues: Editorial and Data Presentation Modifications

### Reviewer #2:

1. In Table 1, the authors show the density of fd Env incorporated in the particles, but what is the density of SIV Env? Was it the same for the low, intermediate and high fd Env viruses? Could the SIV Env density interfere with the recruitment of effector cells after opsonization?

**Authors:** Although we did not quantify the amount of SIV Env incorporated into pseudotyped particles, the Western blot analysis in Fig. 2A indicates that equivalent levels of SIV Env were present in the low, intermediate and high fd Env viruses. Additionally, the similar infectivity levels among each SfdEnv challenge virus (as shown in Fig. 2B) also suggest comparable incorporation of SIV Env.
Due to the lateral mobility of lentiviral Env proteins, interfering of SIV Env with the recruitment of effector cells seems unlikely. Furthermore, our immunoprecipitation assays with PGT121-coated beads suggest that the Fc-portion remains available since successful precipitation of $\mathrm{SfdEnv}^{\mathrm{high}}$ particles was obtained. These aspects are now discussed in the revised manuscript

2. It would be informative if the authors document whether recognition of HIVEnv at the surface of the virus by the complement affects the viral entry mediated by SIVEnv?

**Authors:** We agree that including an *in vitro* assay addressing this matter would be interesting. However, the high complement concentrations needed for such an experiment may impact the viability of TZMbl cells in the assay, making it challenging to determine whether the observed effects are due to HIV Env recognition or simply cytotoxic effects of the complement. Since the animal experiments with the $\mathrm{PGT121}^{\mathrm{LALA\text{-}PG}}$ variant do not indicate a role of complement in sterilizing immunity, we feel that this experiment would not change any conclusions drawn.

### Reviewer #3:

Many of the assays are done twice and “representative” results are shown. Why aren’t results of all the assays shown?

**Authors:** Similar representative experiments are still shown if the absolute values have a large interassay variability, but the effect of the treatment is the same. For Fig. 3B, 3D, 5B and Fig. S3 we now show the mean of all experiments.

Secreted embryonic alkaline phosphatase should not be in caps.
**Authors:** Changed as suggested

1. Figure 2: Though stated elsewhere, it would be helpful to indicate in the legend what the abbreviations for the constructs refer to (e.g., SfdEnvLow = SIVdup pseudotyped with a low quantity of fusion-deficient HIV Env).

**Authors:** Changed as suggested

2. Figure 2A: Can the blot results be quantified?

**Authors:** Although the blots could have been quantified using ImageJ, the results would still be qualitative, as no standard was included in the assay. Therefore, the quantification of Env incorporation into SIVdup particles is not possible due to different staining conditions between each blot. However, the quantitative ELISAs performed highlight the density of fdEnv incorporated into SIVdup pseudotyped particles more accurately.

3. Figure 2D: The figure is difficult to read because of the symbols and colors chosen.

**Authors:** The figure was changed as suggested.

4. Line 310: Should mention that the challenge is with SIVdup pseudotyped with the indicated Envs.

**Authors:** This was modified as suggested.

5. Line 335: Please clarify what is meant by "templates".

**Authors:** The section was rephrased.

6. Line 351: Did that animal have lower serum concentrations of mAb than others? Also, it would be best to show the relevant pharmacokinetic data for each animal in a supplementary figure or table.

**Authors:** The animal #16995 exhibited the second highest PGT121 serum concentration on the day of challenge (10.1 µg/mL), hence within the range of the PGT121-LD-treated animals (5.0 to 14.7 µg/mL). This aspect has been clarified in the Results section.
Supplementary Figure S3 already displayed the PGT121 serum concentrations for each animal before, on the day of, and after the challenge. For clarity, animal #16995 has been highlighted with a distinct color in both Figure 5B and Figure S3.

7. Should mention the rationale for combining blood and lymph node results for the main comparison between groups.

**Authors:** The rationale for combining blood and lymph node results is now provided.

8. The authors claim there is "sterilizing immunity". But all the animals have some virus. Please clarify.

**Authors:** This aspect is now discussed in the revised manuscript.

9. Line 413: the connection between the Hangartner results and the "intrinsic characteristics of each single antibody" is rather vague. Please clarify.

**Authors:** The section was rephrased to address the reviewer´s comment
